# The Membrane-Active Phytopeptide Cycloviolacin O2 Simultaneously Targets HIV-1-infected Cells and Infectious Viral Particles to Potentiate the Efficacy of Antiretroviral Drugs

**DOI:** 10.3390/medicines6010033

**Published:** 2019-02-28

**Authors:** Samantha L. Gerlach, Partha K. Chandra, Upal Roy, Sunithi Gunasekera, Ulf Göransson, William C. Wimley, Stephen E. Braun, Debasis Mondal

**Affiliations:** 1 Department of Biology, Division of Science, Technology, Engineering and Mathematics, Dillard University, New Orleans, LA 70122, USA; 2 Department of Pharmacology, Tulane University Medical Center, New Orleans, LA 70112, USA; pchandr1@tulane.edu (P.K.C.); dmondal@tulane.edu (D.M.); 3 Department of Health and Biomedical Sciences, University of Texas Rio Grande Valley, Brownsville, TX 78520, USA; upal.roy@utrgv.edu; 4 Department of Medicinal Chemistry, Uppsala University, 751 23 Uppsala, Sweden; sunithi.gunasekera@fkog.uu.se (S.G.); ulf.goransson@fkog.uu.se (U.G.); 5 Department of Biochemistry and Molecular Biology, Tulane University Medical Center, New Orleans, LA 70112, USA; wwimley@tulane.edu; 6 Tulane National Primate Research Center, Covington, LA 70112, USA; sbraun@tulane.edu

**Keywords:** cyclotides, cycloviolacin O2, CyO2, HIV-1, protease inhibitors, fusion inhibitors, antiretroviral drugs

## Abstract

**Background:** Novel strategies to increase the efficacy of antiretroviral (ARV) drugs will be of crucial importance. We hypothesize that membranes of HIV-1-infected cells and enveloped HIV-1 particles may be preferentially targeted by the phytopeptide, cycloviolacin O2 (CyO2) to significantly enhance ARV efficacy. **Methods:** Physiologically safe concentrations of CyO2 were determined via red blood cell (RBC) hemolysis. SYTOX-green dye-uptake and radiolabeled saquinavir (^3^H-SQV) uptake assays were used to measure pore-formation and drug uptake, respectively. ELISA, reporter assays and ultracentrifugation were conducted to analyze the antiviral efficacy of HIV-1 protease and fusion inhibitors alone and co-exposed to CyO2. **Results:** CyO2 concentrations below 0.5 μM did not show substantial hemolytic activity, yet these concentrations enabled rapid pore-formation in HIV-infected T-cells and monocytes and increased drug uptake. ELISA for HIV-1 p24 indicated that CyO2 enhances the antiviral efficacy of both SQV and nelfinavir. CyO2 (< 0.5 μM) alone decreases HIV-1 p24 production, but it did not affect the transcription regulatory function of the HIV-1 long terminal repeat (LTR). Ultracentrifugation studies clearly showed that CyO2 exposure disrupted viral integrity and decreased the p24 content of viral particles. Furthermore, direct HIV-1 inactivation by CyO2 enhanced the efficacy of enfuvirtide. **Conclusions:** The membrane-active properties of CyO2 may help suppress viral load and augment antiretroviral drug efficacy.

## 1. Introduction

Since the discovery of human immunodeficiency virus type-1 (HIV-1) as the etiologic agent of acquired immune deficiency syndrome (AIDS), more than 39 million people have died of this epidemic, and alarming annual rates of new infections are estimated at two million people worldwide [1,2]. Despite the availability of numerous antiretroviral drugs (ARVs), only one-third (~36%) of the population living with HIV-1/AIDS have access to expensive medications; therefore, cheaper alternatives, such as natural compounds or phytochemicals, may benefit resource-poor countries [3,4,5]. 

HIV-1 primarily infects and replicates in T-helper cells (Th) and monocytes/macrophages. A number of ARVs are currently available that target different stages of the viral replication cycle. The HIV-1 fusion inhibitors (FIs) suppress virus entry by inhibiting virus (gp120) binding to the cellular receptor (CD4) and coreceptors (CCR5) [6,7,8,9,10]. The cleavage of HIV-1 Gag-Pol polyprotein, carried out by the HIV-1 protease enzyme, is critical in the production of mature virions; thus, the HIV-1 protease inhibitors (PIs) are potent inhibitors of virus maturation and release [11,12,13,14]. Strategies to enhance the efficacy of FIs and PIs are clearly needed. In this respect, since the infectious HIV-1 virions assemble at the plasma membrane ‘lipid-rafts’ followed by virus exit from cells carrying a lipid envelope that is similarly enriched with phospholipids [15,16] the lipid membrane of infected cells and the lipid envelope of the released virion are novel targets. 

Although cART is initially very effective in suppressing plasma viral load (PVL), it may eventually fail due to non-adherence to the treatment regimen and, to a lesser extent, compromised in vivo pharmacokinetics (PK) [17,18,19]. Increased expression of drug-efflux pumps has been associated with decreased intracellular ARV levels, and long-term treatment with higher doses of ARV increases side effects and decreases patient compliance, which facilitates the resurgence of multidrug-resistant (MDR) viruses [20,21,22]. Therefore, new antiviral agents that overcome current MDR mechanisms, and target either the productively infected cells or the infectious virions, will be of crucial importance [23,24].

Studies have identified numerous plant-derived compounds that exhibit anti-HIV activity [25,26]. Interestingly, the largest collections of natural products that display potent antiviral activity are from the family of phytopeptides known as cyclotides [27,28,29,30]. These gene-encoded peptides are approximately 25–37 amino acid residues long and are characterized by a unique cyclic cystine knot topology including three disulfide bonds. Several of these cyclic peptides have potent antimicrobial effects, show remarkable stability in vivo, and have a novel target against microbes, i.e., the lipid membrane [30,31]. Although recent mouse competition experiments indicate that resistance to cycloviolacin O2 (CyO2) can evolve through several mutations with only minor fitness costs [32], a targeted lipid membrane mechanism of action may still explain why some cyclotides evade the resistance mechanisms frequently seen with ARVs. Therefore, studies on the in vivo bioactivity of cyclotides warrants further attention as novel adjuvant agents in HIV-positive individuals on combination ARV therapy (cART). 

**Figure 1:**
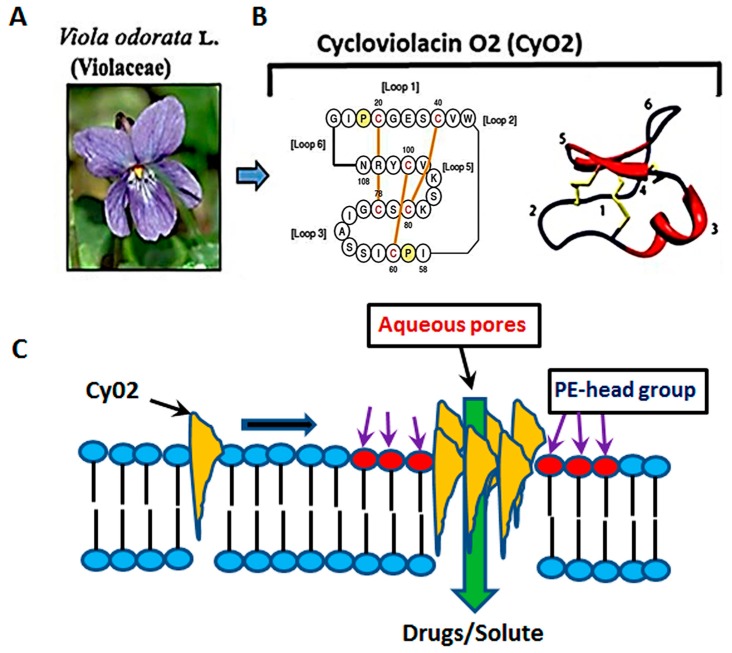
The membrane-active properties of CyO2. (**A**) CyO2 was isolated and purified from the leaves of *Viola odorata* L. (Violaceae). (**B**) Amino acid sequence and the 3-D structure of this cyclic peptide are shown. (**C**) A schematic of the membrane targeting, multimerization and pore-formation ability of cyclotides. CyO2 has an affinity for ‘raft-like’ membrane microdomains that are rich in phosphatidyl ethanolamine (PE).

Numerous past studies have suggested that the anti-HIV activity of the cyclotide, kalata B1 (kB1) is dependent upon the lipid composition of target cell membranes [30,31,33]. Henriques et al. (2011), demonstrated that the insertion of kB1 occurs at regions rich in phosphatidyl ethanolamine (PE), where membrane pore-formation occurs due to facilitated peptide-to-lipid hydrophobic interactions and their subsequent multimerization [30]. However, high concentrations (~10 μM) of kB1 were used to inactivate the HIV-1 virus, which caused cytotoxic effects and significant hemolysis [31,33] dampening their therapeutic potential. Several other cyclotides, such as cycloviolins A–D, also displayed anti-HIV-1 activities [34,35]. However, similar to kB1, doses used to show antiviral effects were cytotoxic. Thus, despite the apparent successes in isolating cyclotides with anti-HIV properties, their utility as drug leads has been significantly hindered. Furthermore, although it is postulated that different cyclotide members may recognize different membrane constituents [36,37,38], their ability to preferentially target the membrane of HIV-1-infected cells or viral particles have not been thoroughly studied. In this respect, studies using CyO2, a prototypic cyclotide extracted from the leaves of the sweet violet (*Viola odorata* L. Violaceae), showed its potent pore-forming ability and exceptional bactericidal effects [32,38] (Figure 1A,C). 

Although the suboptimal antimicrobial activity of cyclotides was due to their lack of ability in targeting the bacterial cell, the positively charged CyO2 (+2) showed an affinity for the negatively charged outer membrane of *Escherichia coli* (*E. coli*) [31,39,40]. However, despite several past observations that the membranes of both HIV-1-infected cells [15,16,41] and envelopes of viral particles are similarly enriched with ‘raft-like’ lipid microdomains [42,43], the membrane-targeting efficacy of CyO2 in enabling its potent anti-HIV effects has not been well established. In previous studies, using a model of chronic infection in a monocytic cell line (U1), we were the first to document the potent anti-HIV effects of CyO2 [44]. The current study provided evidence that CyO2 has potent anti-HIV effects in several T-lymphocyte models [HuT78*, HIV IIIB H9 and J-Lat (9.2)] as well, and in vitro findings showed that CyO2, at doses that do not cause hemolysis of human red blood cells (RBC) can still impact the uptake and antiviral efficacy of anti-HIV agents, both saquinavir (SQV) and nelfinavir (NFV). In addition, these findings demonstrated that CyO2 can disrupt the integrity of viral particles to suppress their infectivity, and this direct anti-HIV effect enabled CyO2 to increase the efficacy of the entry inhibitor drug, enfuvirtide (T-20). Therefore, the membrane-active properties of CyO2 may be a novel adjuvant in HIV-1-positive patients on cART.

## 2. Materials and Methods

### 2.1. Tissue Culture

The following five cell lines were obtained from the AIDS Research and Reference Reagent Program (ARRRP): (i) HIV IIIB H9, a chronically infected T-cell line; (ii) U1, a chronically infected monocytic line; (iii) J-Lat(9.2), a Jurkat T-cell line where activation of reporter (GFP) is regulated by HIV-1 LTR; (iv) TZM-blue, a luciferase (Luc) reporter line used to measure viral infectivity, and (v) PM1, a HIV-1 infectable T-cell line. Another T-cell line, HuT78 and a promonocytic line, U937 were also purchased from American Type Culture Collection (ATCC, Manassas, VA). All the above cell lines were cultured in RPMI-1640 media containing 10% heat-inactivated fetal bovine serum (FBS) and 1% penicillin/streptomycin (Sigma-Aldrich, St. Louis, MO, USA). Cells were passaged every 3–4 days at a 1:6 dilution and replenished with complete growth media. The primary brain endothelial cells (hBMVEC) and culture media were obtained from Cell Systems (CS-C) (Kirkland, WA) and cultured according to the supplier’s recommendations. Experiments with hBMVECs were conducted within cell passages 4–6. All of the above cell types were grown in a 37 °C incubator with 5% CO_2_. Type O+ single donor human red blood cells (IPLA-WB3-25490) were obtained from Innovative Research Inc. (Novi, MI, USA).

### 2.2. Reagents

CyO2 was isolated from the leaves of sweet violet (*Viola odorata* L. Violaceae) and HPLC purified according to our previously published studies [45]. Melittin (MEL), a positive pore-former isolated from bee venom, was purchased from Invitrogen (Eugene, OR). Both CyO2 and MEL powders were stored at 4 °C in a desiccated glass vial. Solutions were prepared as 1.0 mM stocks in absolute ethanol (Sigma-Aldrich), stored at −20 °C and diluted to appropriate concentrations before each experiment. The PIs, saquinavir (SQV) and nelfinavir (NFV) and the FI, enfuvirtide (T20) were obtained from the ARRRP, NIH (≥ 98% HPLC grade). Both SQV and NFV were prepared as 1 mM stocks in dimethyl sulfoxide (DMSO) and T-20 solutions were prepared as a 1 μM stock in phosphate buffered saline (PBS). Stocks were stored at −20 °C and diluted in media before each experiment. 

### 2.3. Preparation of CFV and VLP Stocks

Cell-free virus (CFV) was harvested from the supernatants of HIV IIIB H9 cells by filtration (0.45 µm) to remove cell debris and ultracentrifugation (100,000× *g*) to pellet the virus. In each batch, viral titers were determined by HIV-1 p24 ELISA and the multiplicity of infection (MOI) calculated according to our past publications [7,44]. All CFV stocks were stored at −80 °C and thawed on ice before experiments. In addition, the HIV-1-based virus-like particles (VLP) were generated by a three plasmid transient transfection method using calcium phosphate [46]. The three plasmids were, (i) pHR.cmv-eGFP.ST, (ii) pCMVΔR8.91, and (iii) pJRFL. Conditioned media were collected at 72 h post-transfection and VLPs were concentrated using the PEG-it™ virus precipitation solution (System Biosciences, Palo Alto, CA, USA), aliquoted and stored at −80 °C. 

### 2.4. HIV-1 Infection and Treatment Protocols

For acute infection in T-cells, the PM1 or HuT78 cells were exposed to virus stocks (MOI~2) from HIV IIIB H9 cells (HTLV-IIIB strain) for 24 h, following which cells were washed with PBS, re-suspended in complete growth media, in presence or absence of treatments. At specified time points (3, 5 or 7 days), viral titers were measured in culture supernatants by p24/Gag ELISA. Cells were exposed to the pore-formers (CyO2 and MEL) and/or the ARVs (SQV, NFV or T-20) for specified time points and changes in HIV-1 p24 were measured. For experiments using chronically infected U1, cells were first stimulated with PMA (100 ng/mL for 2 h), washed with PBS and cultured in complete growth media in presence or absence of treatments (ARVs and/or CyO2). 

The effects of CyO2 on efficacy of HIV-1 PIs (SQV or NFV) were determined by first exposing the infected cells to CyO2 for 30 min, followed by the addition of SQV or NFV for either short-term (2–6 h) or following long-term (72 h) exposure, removal of drugs and viral titer measurements (p24 ELISA) at the specified times. The effects of CyO2 on the inhibitor, T-20 were measured by pre-treatment of uninfected PM1 cells with T-20 for 2 h, followed by exposure to HTLV-IIIB virus for 24 h, removal of the virus, growth in complete media and p24 determination at 3- and 6-days. The effects of CyO2 on virus disruption were measured by pre-exposing CFV stocks (HTLV-IIIB strain) to CyO2 for 2 h, followed by ultracentrifugation for 1 h to pellet the intact virions and measurement of viral p24 content. The direct effects of CyO2 on viral infectivity were tested by exposure of CFV stocks to increasing doses of CyO2 for 2 h, 10-fold dilution in media, and then addition of virus to cells and measurement of p24 levels at specified time points.

### 2.5. Hemolytic Assays

To assess the hemolytic activity of CyO2 concentrations (0.05, 0.10, 0.25, 0.5, 1, 2.5, 5.0, and 10.0 μM), human red blood cells (1 × 10^8^ per treatment) were incubated with human serum (HS) or fetal bovine serum (FBS) for 1 h and 18 h, after which the Synergy/HTX Multimode Reader and Gen 5 3.02 software was used to analyze absorbance data. Briefly, in addition to negative (PBS) and positive (TritonX 100) controls, the human red blood cells were treated with CyO2 concentrations and human serum or FBS for 1 h. Treatments were centrifuged at 1000× *g* for 2 min and samples photographed. To obtain Synergy/HTX Multimode absorbance measurements, 25 μL of treatment supernatant was mixed with 75 μL of PBS. The remaining re-suspended pellets were incubated for 17 h at 37 °C prior to photographing and additional absorbance measurements.

### 2.6. SYTOX Green Uptake Assays 

The fluorescent nucleic acid stain, SYTOX Green (Invitrogen, Eugene, OR, USA) was used to measure CyO2-induced pore-formation in both uninfected and HIV-infected cells [43]. Briefly, cells (1 × 10^5^ per well) seeded into 96-well flat bottomed microtiter plates, were treated with either CyO2 (0.5–1.5 µM) or Melittin (5 µM) in a solution containing SYTOX Green (0.04 µM) for 1–30 min, along with intermittent shaking. Fluorescence measurements were taken at 1 min intervals using a MicroPlate Reader with Tungsten light source (485 nm excitation and 530 nm emission wavelengths). Pore-formation, as measured by average fluorescence of treated wells relative to control wells, were normalized to the fluorescence in untreated wells (0%) and in wells exposed to Melittin (100%). Percent membrane leakage observed with increasing doses of CyO2 was then calculated.

### 2.7. ^3^H-SQV Uptake Assays

Radio-labeled ^3^H-SQV (specific activity: 1.0 Ci/mM) was obtained from Moravek Biochemicals (Brea, CA, USA) and was used to measure intracellular drug accumulation [7]. Briefly, HuT78 cells were cultured in 24-well plates (5 × 10^5^ per well) and pre-exposed to CyO2 (0.5 and 1.5 μM) for either 10 min or 30 min, followed by washing off with PBS and addition of ^3^H-SQV (1.7 pM) and incubation at 37 °C for 2 h. Cells were harvested by centrifugation and extracts obtained by lysing with 1.0 M ammonium hydroxide (NH_4_OH). Intracellular levels of ^3^H-SQV were monitored in the lysates, and 100 μL of the lysate was used to measure protein levels by using the BCA protein assay kit (ThermoFisher, Waltham, MA, USA). The remaining 100 μL of the lysate was dissolved in 10 mL of EcoLite scintillation fluid from MP Biomedicals (Santa Ana, CA, USA) and count per minute (CPM) were determined by using a Tri-Carb 2800TR Liquid Scintillation counter (Perkin Elmer, Waltham, MA, USA). In respective samples, data were normalized to the protein contents and presented as CPM/μg of protein. 

### 2.8. Enzyme-Linked Immunosorbent Assays

Viral titers were measured using an HIV-1 Gag/p24 enzyme-linked immunosorbent assay (ELISA) kit from Advanced BioSciences (Kensington, MD, USA) and according to the manufacturer’s instructions [44]. In each experiment, cells were centrifuged and aliquots of supernatants were transferred to the activated ELISA plates. Briefly, plates were washed with wash buffer (300 µL/ well) and then with the disruption buffer (25 µL/well). Supernatants (100 µL each) were then added to wells, covered with plate sealer and incubated for 1 h at 37 °C. After incubation, samples were washed (4 times) and 100 µL of conjugate solution added, plate sealed and incubated at 37 °C for another for 1 h. Following another round of washing, 100 µL of the peroxidase substrate was added to each well (in the absence of light) and incubated at 37 °C for 30 min. The reaction was stopped by using 100 µL of the stop solution, and absorbance quantified using a Maltiskan Plus Spectrophotometer (450 nm wavelength). In each plate, both positive p24 standards and negative control wells were also included. Change in HIV-1 p24 level (pg/mL) was calculated using linear regression.

### 2.9. HIV-1 LTR Reporter Assays

Two GFP-reporter lines, U937-VRX494 and the J-Lat (9.2) were used to measure the effect of CyO2 on HIV-1 long terminal repeat (LTR) function under both basal and PMA-stimulated conditions. The U937-VRX494 cells were generated by stably transducing U937 monocytic cells with an HIV-based lentivirus construct expressing GFP under the control of HIV-LTR (VRX494) [47]. In each experiment, cells were cultured with CyO2 (0.25–1.0 μM) and/or PMA (5 ng/mL) for 72 h, and GFP expression was monitored by both fluorescence microscopy and flow cytometry. Both the number of GFP-positive cells as well as their mean fluorescence intensity (Mean FITC-A) were determined by using a FACScan flow cytometer from Beckman-Coulter (LCRC core facilities, Tulane University, New Orleans, LA, USA). 

### 2.10. Infectivity Assays Using TZM-bl Cells

Luciferase reporter assays were carried out to measure the viral infectivity using the TZM-bl cell line [48]. Briefly, cells (2 × 10^4^/well) were cultured in a 24-well plate one day before the experiment. The CFV stocks were pre-exposed to CyO2 (0.25–5.0 μM) for 2 h at 37 °C. Before the addition of CFV to the TZM-bl cells, the final concentrations of CyO2 were diluted 10-fold (to 0.25 μM and 0.5 μM respectively). Cells were exposed to CFV for 72 h, followed by washing with PBS, lysing with 200 μL of 1× lysis buffer, centrifugation (5000 rpm, 5 min) and measurement of Firefly luciferase activity using a Dual-luciferase assay kit from Promega (Madison, WI, USA). Cells exposed to untreated CFV were used as controls. The relative light units (RLU) obtained for both control and CFV-exposed cells were determined by using SYNERGY/HTX Multi-mode reader from BioTek (Winooski, VT, USA). 

### 2.11. Statistical Analysis

Data are representative of at least three independent experiments carried out in at least triplicate samples. Error bars represent the mean ± standard deviations (SD). All data were analyzed with GraphPad Prism 5.0 software (GraphPad, San Diego, CA, USA) using a combination of two-tailed Student’s t-test, analysis of variance (ANOVA), Bonferroni’s multiple comparisons, and Dunn’s multiple comparisons. A *p*-value of less than 0.05 was considered statistically significant. 

## 3. Results

### 3.1. Low CyO2 Concentrations Do Not Cause Significant Hemolytic Activity on Human Red Blood Cells

**Figure 2:**
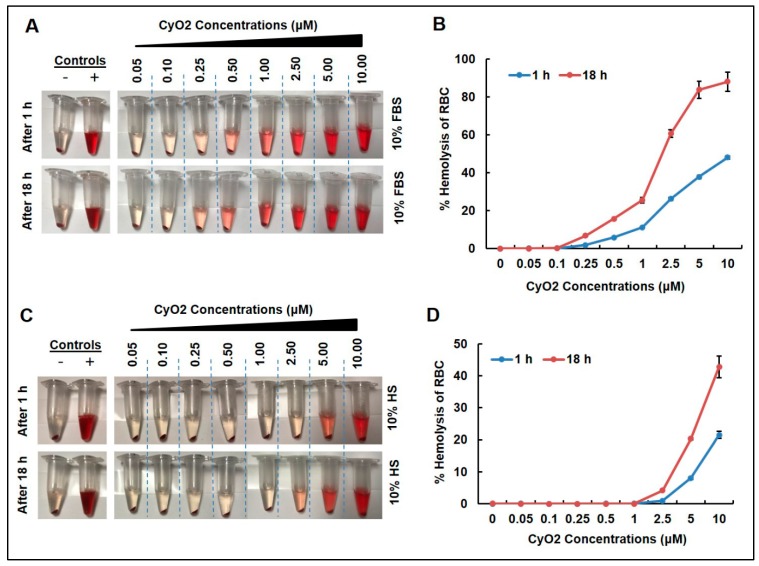
Time and dose-dependent effects of CyO2. CyO2 demonstrated dose-dependent hemolytic activity in red blood cells incubated for 1 and 18 h, in the presence of either fetal bovine serum (FBS) (**A**,**B**) or human serum (HS) (**C**,**D**). Error bars show +/- standard error of means (SEM).

To address the fact that clinical research with naturally occurring phytopeptides has been hindered by their hemolytic activity [31,33], the effect of CyO2 on human red blood cells (RBC) incubated with human serum or FBS was measured. As illustrated in Figure 2, concentrations of CyO2 (0.05–10 μM) caused visible RBC lysis in a dose-dependent manner when incubated with FBS (Figure 2A) and human serum (HS) (Figure 2C) for 1 and 18 h. The percentage of hemolytic activity on RBCs was generally lower in samples incubated with HS compared to FBS (Figure 2B,D). Concentrations of CyO2 (0.05–2.5 μM) incubated with FBS exhibited less than 60% hemolytic activity (Figure 2B). CyO2 concentrations (0.05–2.5 μM) demonstrated less than 10% hemolytic activity in samples incubated for 1 or 18 h with HS (Figure 2D). Furthermore, CyO2 (< 1 μM) exhibited no hemolytic activity when incubated with HS. Therefore, the remainder of this research primarily evaluated CyO2 concentrations ≤ 1.5 μM. 

### 3.2. Non-Hemolytic Concentrations of CyO2 Retain Its Pore-Forming Ability and Induce Greater Pore-Formation in HIV-Infected Cells

**Figure 3:**
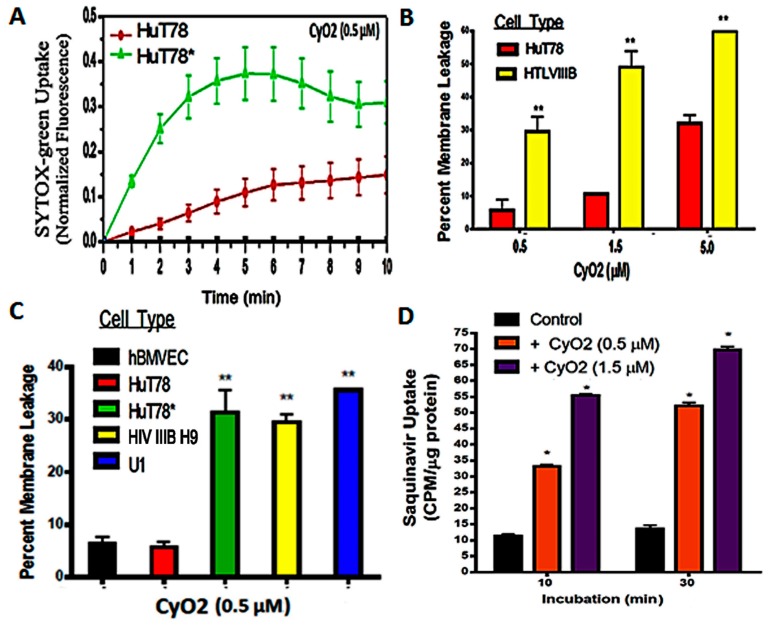
Effect of CyO2 on pore-formation and drug-uptake in HIV-infected cells. (**A**) Fluorimetric analysis of SYTOX-green uptake in uninfected HuT78 cells and HIV-infected HuT78* cells demonstrate temporal (0–10 min) changes in SYTOX-green fluorescence following exposure to CyO2 (0.5 μM). (**B**) Dose-dependent effects of CyO2 (0.5–5.0 μM) on SYTOX-green uptake in uninfected HuT78 and chronically-infected HIV IIIB H9 cells. (**C**) Comparative analysis of SYTOX-green uptake in primary hBMVEC cells, uninfected HuT78 cells, and productively-infected cells, HuT78* and HIV IIIB H9 cells, at 10 min post-exposure to CyO2 (0.5 μM). Percent change in membrane leakage in CyO2-exposed cells, as compared to untreated control (0%) and in cells exposed to Melittin (100%) are shown. (**D**) Effect of CyO2 pre-exposure on radiolabeled saquinavir (^3^H-SQV) uptake in HuT78 cells. Counts per minute (CPM) values were normalized to protein contents (CPM/μg protein). Non-hemolytic doses of CyO2 induced more pore-formation in productively-infected cells, which increased both SYTOX-green and ^3^H-SQV uptake. (*, *p* < 0.05; **, *p* < 0.01)

SYTOX Green-dye uptake assays demonstrate that non-hemolytic CyO2 concentrations retain its membrane-disrupting ability. Furthermore, the optimal dose (<1.5 μM) and time (<6 h) of CyO2 exposure were used for its anti-HIV effects in the remaining studies (Figure 3). CyO2 has an affinity for lipid-raft membrane microdomains [38,40], especially those rich in PE-rich lipids [39,49,50]. Similarly, lipid constituents are found on the plasma membranes of productively infected cells [15,43]. The current work investigated whether CyO2 shows a preferential ability to target the HIV-infected vs. uninfected cells (Figure 3A–D). 

SYTOX Green nucleic acid stain is impermeable to an intact plasma membrane but rapidly internalizes via membrane pores [44]. Therefore, temporal changes in SYTOX Green uptake following CyO2 (0.5 and 1.5 μM) exposures were compared in both uninfected HuT78 and in the acutely-infected HuT78* cells. Melittin (5.0 μM) was used as a positive control (100% pore formation) and untreated cells were negative controls (0% pore formation). Increases in intracellular fluorescence were evident within minutes following CyO2 exposure. Interestingly, the HIV-infected HuT78* cells showed 3–4 fold higher SYTOX Green fluorescence than the uninfected HuT78 cells (Figure 3A). Furthermore, a comparative analysis in the chronically-infected HIV IIIB H9 cells and the uninfected HuT78 cells also demonstrated this differential effect on pore-formation and dye uptake (Figure 3B). Most differences in pore-formation was apparent at the lower doses of CyO2, and significant differences were seen between uninfected cells, i.e., hBMVECs and HuT78 cells, and the HIV-infected cells, i.e., HuT78*, HIV IIIB H9 and U1 cells (Figure 3C). Findings indicated that non-hemolytic doses of CyO2 can rapidly disrupt the membranes in HIV-infected cells. 

### 3.3. CyO2 Facilitates Intracellular Uptake of Radiolabeled Saquinavir

We previously documented that non-hemolytic doses of CyO2 can enhance the uptake of doxorubicin in breast cancer cells [45]. The research described in this publication investigates whether CyO2-mediated pore-formation can be similarly exploited to increase the uptake of anti-HIV drugs and increase their anti-HIV efficacy. The cellular entry of large molecular weight drugs like PIs remains a significant challenge [18]; therefore, this study tested whether CyO2 can increase the intracellular uptake of tritium-labelled saquinavir (^3^H-SQV). HuT78 cells were first exposed to CyO2 (0.5 and 1.5 μM) for 10–30 min, washed off, and then incubated with ^3^H-SQV for 2 h prior to harvest. Intracellular ^3^H-SQV was measured by scintillation counting of cell lysates and normalization of counts per minute values (CPM/μg protein). As compared to cells exposed to ^3^H-SQV alone, which showed negligible intracellular SQV levels after 2 h incubation, significantly higher ^3^H-SQV uptake was documented in the CyO2 pre-treated cells. Therefore, pore-formation by non-hemolytic doses of CyO2 increases SQV uptake (Figure 3D).

### 3.4. Co-Exposure to CyO2 Impacts the Anti-HIV Efficacy of Multiple Protease Inhibitors

**Figure 4:**
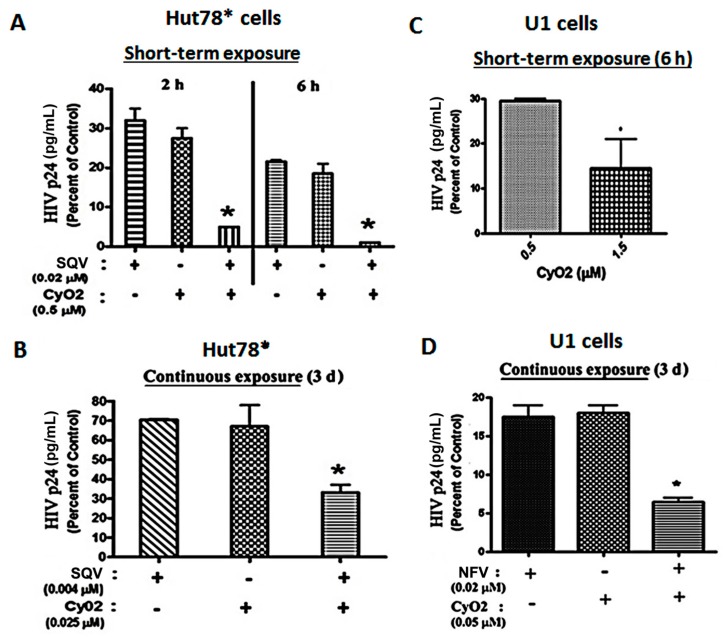
Anti-HIV effects of CyO2, alone and in combination with PIs. In (**A**,**B**), the effects of CyO2 and/or SQV is shown in HIV-1 infected HuT78* cells. HIV-1 p24 levels at 3-days post-infection, following either (**A**) short-term (2–6 h) exposure to SQV (0.02 μM) and/or CyO2 (0.5 μM) or (**B**) following continuous exposure (72 h) to SQV (0.004 μM) and/or CyO2 (0.025 μM), are shown. In (**C**,**D**), the effects of CyO2 and/or nelfinavir (NFV), in HIV-1 infected U1 cells are shown. The p24 levels at 3-days, following either (**C**) short-term (6 h) exposure to CyO2 (0.5 and 1.5 μM); (**D**) continuous exposure (72 h) to NFV (0.02 μM) and/or CyO2 (0.05 μM) are shown. Bar graphs show normalized HIV-1 p24 levels (pg/mL) in culture supernatants and are represented as a percent of control (untreated cells). Error bars show +/- standard error of means (SEM) from 3–4 independent experiments. Significant changes from control are shown as *p*-values (*, *p* < 0.05). Exposure to CyO2 alone decreased virus production and coexposure to CyO2 increased the anti-HIV efficacy of two different PIs, SQV and NFV.

In both acutely-infected T-cells (HuT78*) and chronically-infected monocytic cells (U1), the ability of CyO2 to augment the efficacy of two PIs, SQV and NFV, was investigated (Figure 4A–D). In short-term exposure studies, HuT78* cells were exposed to SQV (0.02 μM) and/or CyO2 (0.5 μM) for either 2 h or 6 h, followed by removal of drugs, and culturing of cells for 3-days and measurement of HIV-1 p24 in culture supernatants (Figure 4A). Even short-term exposure to CyO2 and SQV significantly reduced HIV p24 content. Almost a total suppression of HIV-1 p24 was seen following exposure to this combination for 6 h. Interestingly, short-term exposure to CyO2 alone also showed a 70%–80% reduction in HIV p24 levels, which implicated its direct effect on the virus as well (Figure 4A). In addition, Figure 4B shows that continuous exposure to much lower concentrations of SQV (0.004 μM) and CyO2 (0.025 μM) was able to suppress HIV p24 production by the HuT78* cells. Notably, these very low concentrations of SQV or CyO2 alone did not significantly inhibit HIV-1 replication (~20%–30%); however, coexposure enabled a remarkable suppression (~70%–80%) in HIV-1 p24 levels.

Similar studies were carried out in the U1 cells, which were first stimulated with PMA (10 ng/mL) in order to activate virus production. In these cells, the effect of CyO2 alone and in conjunction with NFV was investigated (Figure 4C,D). Short-term exposure studies (6 h) showed that increasing CyO2 concentrations decreased HIV-1 p24 levels, where 0.5 μM of CyO2 caused ~70% suppression and 1.5 μM of CyO2 caused as much as an 85%–95% suppression (Figure 4C). Moreover, continuous exposure studies (72 h to lower concentrations of CyO2 (0.05 μM) and/or NFV (0.02 μM)) inhibited 95% of HIV-1 p24 production by the PMA-stimulated U1 cells (*p* < 0.05) (Figure 4D). These in vitro findings showed that CyO2 can increase the efficacy of two different PIs, i.e., SQV and NFV. Most interestingly, our research also suggested that non-hemolytic doses of CyO2 alone might also have a direct anti-HIV effect. 

### 3.5. CyO2 Does Not Alter HIV-1 LTR Function

**Figure 5:**
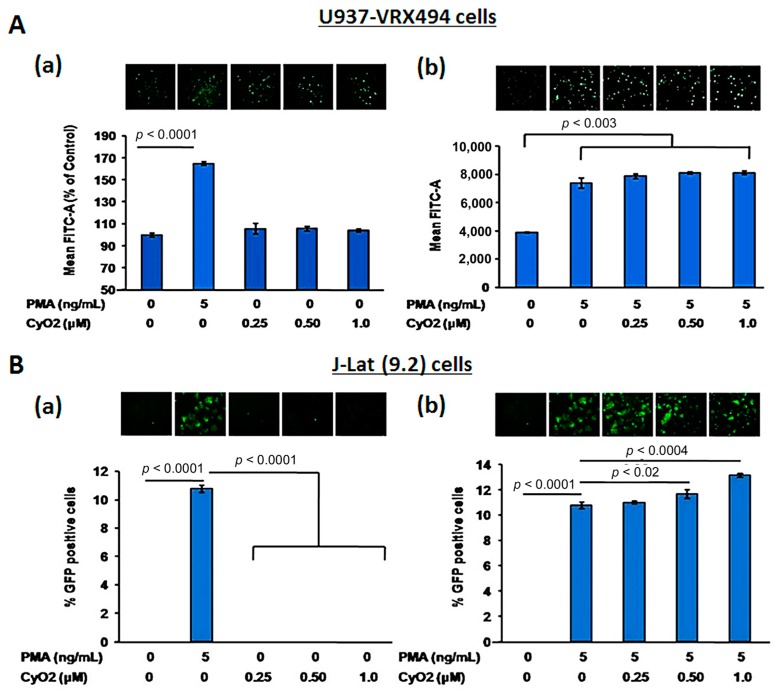
Effect of CyO2 on HIV-1 LTR function under unstimulated and PMA-stimulated conditions. In (**A**), the effect of CyO2 (0.25–1.0 μM) on HIV-1 LTR directed GFP expression from U937-VRX cells are shown. Top panels show a representative image of GFP expressing cells and bottom panels show percent change in mean fluorescence (FITC-A). In (**B**), the effect of CyO2 (0.25–1.0 μM) on HIV-1 LTR directed GFP expression from J-Lat (9.2) cells are shown. Top panels show a representative image of GFP expressing J-Lat (9.2) cells and the percent change in GFP-positive cells is shown in the bar graphs. Error bars show +/-SEM of two independent experiments in triplicate samples. Significant changes from controls are shown as P-values (*, *p* < 0.05). CyO2 did not alter HIV-1 LTR function in either U937-VRX (pro-monocytic) or J-Lat (9.2) (T-lymphocytic) cell lines.

To further investigate the potential CyO2 mechanism of action and to ascertain whether the anti-HIV effects of CyO2 may be due to its suppressive effects on HIV-1 LTR function, two GFP-reporter cell lines, U937-VRX and J-Lat (9.2) were used (Figure 5A,B). Both U937-VRX (Figure 5A) and J-Lat (9.2) (Figure 5B) were exposed to increasing concentrations of CyO2 (0.25–1.0 μM) in the absence (panel-a) or presence (panel-b) of PMA (5 ng/mL). The HIV-1 LTR-directed GFP expression was then measured by flow cytometry after 48 h. Results in U937-VRX cells indicated that CyO2 does not alter basal HIV-1 LTR function, and neither did it suppress the PMA-stimulated HIV-LTR activation (Figure 5A). Similarly, the flow cytometry analysis of GFP-positive J-Lat (9.2) cells showed that CyO2 does not change basal LTR function or prevent the PMA-stimulated LTR activation (Figure 5B). Therefore, the effect of CyO2 on HIV-1 p24 production is not due to an effect on HIV-1 LTR.

### 3.6. CyO2 Suppresses the Infectivity of Viral Particles

**Figure 6:**
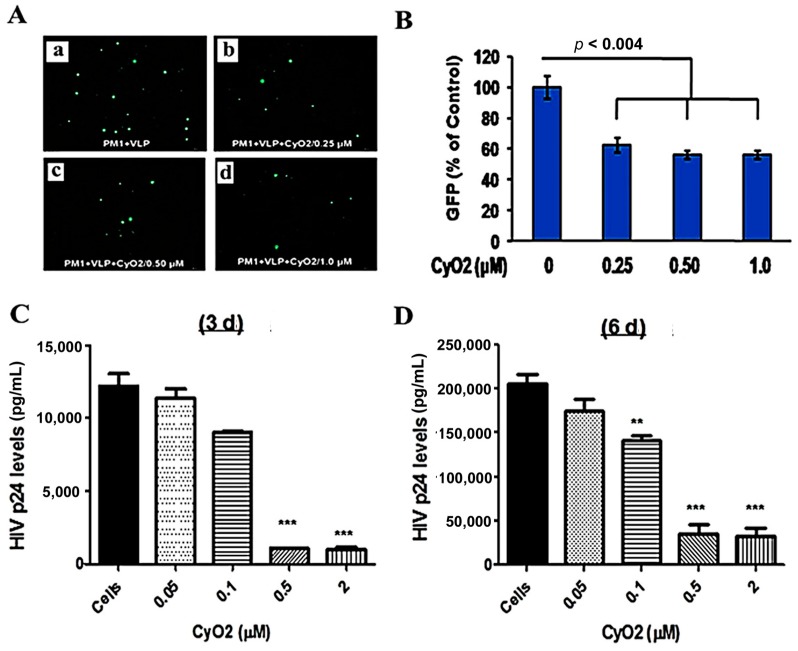
Effect of CyO2 on infectivity of viral particles. In (**A**,**B**), PM1 cells were incubated with untreated (control) or CyO2 (0.25–1.0 μM) treated virus-like particles (VLP) and changes in GFP expressing cells were measured after 48 h. A representative image of GFP-positive cells is shown in (**A**) and percent change in GFP-positive cells is shown in (**B**). Pre-exposure to CyO2 decreases the infectivity of VLPs. In (**C**,**D**), the HTLVIIIB cell-free virus (CFV) were exposed to CyO2 (0.05–2.0 μM) for 2 h prior to incubation of PM1 cells. HIV-1 p24/Gag levels in culture supernatants were measured after 3-day (**C**) and 6-day (**D**). Exposure of CFV to CyO2 significantly (*p* < 0.001) decreased their infectivity in PM1 cells. (**, *p* < 0.01; ***, *p* < 0.001)

The envelopes of HIV-1 particles are enriched with lipid raft constituents [15,43,51], which may be preferentially targeted by CyO2. To investigate this novel phenomenon, the direct effect of CyO2 on infectious viral particles was measured (Figure 6A–D). Initial studies were carried out using an attenuated HIV-1 based virus-like-particle (VLP) that enables direct measurement of infected cells by GFP microscopy and flow cytometry (Figure 6A). For these studies, VLPs were pre-exposed to CyO2 (0.25–1.0 μM) for 2 h, followed by their incubation with PM1 cells and measurement of their infectivity by enumerating GFP expressing cells. As compared to PM1 cells exposed to the CyO2 pre-treated VLPs, the PM1 cells exposed to control VLPs showed a much higher number of GFP-expressing cells after 48 h. Decreases in the number of GFP-expressing cells are shown in the representative image (Figure 6A) and in the bar graphs (Figure 6B). Results from this single-round infection model (VLP) provided the first evidence that short-term exposure to sub-toxic doses of CyO2 can have a profound effect in suppressing the infectivity of HIV-1 particles. 

To further confirm these findings, the infectious HTLV-IIB virus was exposed to concentrated stocks of CyO2 (0.05–2.0 μM) for 2 h, followed by 10-fold dilution of the solution to suppress any cytotoxic effects, and then infection of PM1 cells. Virus production by PM1 cells, exposed to either untreated HTLV-IIB (control) or CyO2-treated HTLV-IIIB (experimental) were measured by HIV-1 p24 ELISA after 3-days and 6-days (Figure 6C,D). Congruent with the GFP-data obtained with VLPs, pre-exposure to CyO2 significantly decreased the p24 production by the HTLV-IIIB infected PM1 cells. Interestingly, in comparison to the effect on VLPs, the suppressive effect of CyO2 on replication competent virus showed a more dramatic suppression.

### 3.7. CyO2 Disrupts Viral Particles, Decreases Their Infectivity in the TZM-bl Model, and Increases the Anti-HIV Efficacy of Enfuvirtide

**Figure 7:**
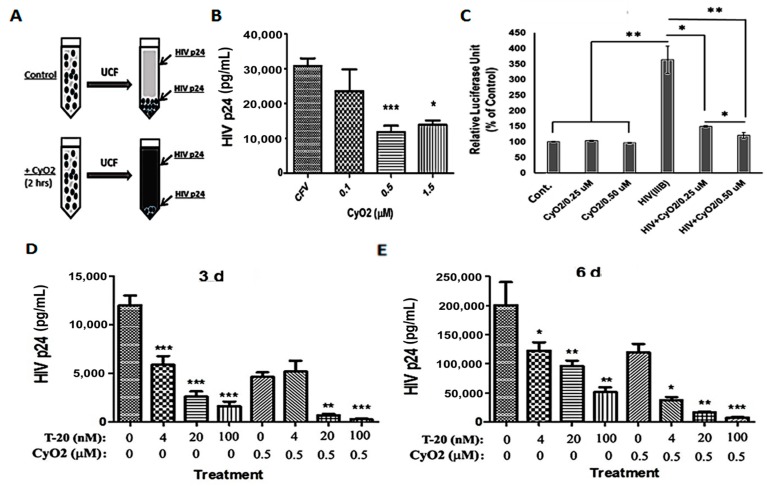
Direct effect of CyO2 on viral particle disruption and T-20 efficacy. (**A**) A schematic of CyO2 exposure, ultracentrifugation (UCF) of CFVs, and measurement of p24 content in viral pellets. (**B**) Effect of 2 h exposure of HTLV-IIIB to CyO2 (0.1–1.5 μM) followed by UCF, and determination of p24 content. CyO2 rapidly disrupts viral particles and decreases their internal p24 contents. (**C**) Effect of CyO2 (0.25 and 5.0 μM) on viral infectivity using the reporter cell line, TZM-bl. Relative luciferase units (RLU) values show that pre-exposure to CyO2 decreases the infectivity of HTLV-IIIB. In (**D**,**E**), PM1 cells were exposed to untreated (control) or CyO2 (0.5 μM) pre-treated HTLV-IIIB virus and then added to cells pre-exposed (2 h) to enfuvirtide (T-20; 4–100 nM). Changes in HIV-1 p24 (pg/mL) in culture supernatants were measured after 3-day (**D**) and 6-day (**E**). CyO2 enhances the efficacy of T-20. (*, *p* < 0.05; **, *p* < 0.01; ***, *p* < 0.001)

Initial studies were carried out to see whether the membrane-targeting ability of CyO2 can disrupt the integrity of viral particles and decrease their internal contents (Figure 7A–E). For these studies, concentrated HTLV-IIIB stocks were first incubated with CyO2 (0.1–1.5 μM) for 2 h, followed by ultracentrifugation (100,000× *g*), removal of the supernatants, and determination of p24-levels in the viral pellets (Figure 7A). We observed that short-term exposure (2 h) to low-dose CyO2 (0.1–1.5 μM) markedly decreased HIV p24 levels in the viral pellets, which indicated the disruption of intact viral particles by CyO2 (Figure 7B). To further confirm that CyO2-induced viral disruption can decrease infectivity, a highly quantitative HIV-infectivity assay was employed by using the TZM-bl cell line [48]. Percent change in relative luciferase units (RLU) clearly showed a 4-fold increase in cells exposed to HTLV-IIIB virus. However, no significant increase in luciferase activity was seen when the TZM-bl cells were exposed to the CyO2-treated virus (Figure 7C). Indeed, studies on the effects of CyO2 on viral particles, which showed decreased HIV p24 levels; and the TZM-bl assay, which showed decreased LTR-directed luciferase expression (RLU) established the direct suppressive effect of CyO2 on infectious viral particles. 

The HIV-1 fusion inhibitor T-20 suppresses viral entry [10]. Since CyO2 can directly suppress viral infectivity, we investigated whether CyO2 can also be used to enhance the antiviral efficacy of T-20. Non-hemolytic doses of CyO2 (0.5 μM) alone and in combination with T-20 (4–00 nM) were used to document their anti-HIV effects in HIV-infected PM1 cells (Figure 7D,E). The HIV-1 p24 ELISA results were analyzed after 3-days (Figure 7D) or 6-days (Figure 7E). Measurements of p24 levels at 3 and 6-days post-infection clearly indicated that co-exposure to CyO2 increases the anti-HIV efficacy of T-20. Although T-20 alone suppressed infectivity by 3-5 fold, coexposure to CyO2 more significantly abrogated p24 production (*p* < 0.001). 

## 4. Discussion

This study provides the first evidence that non-hemolytic concentrations of CyO2 may be used as a novel strategy to directly disrupt the HIV-1 particles. In addition, these studies show that the rapid pore-formation by CyO2 may be used to increase the therapeutic efficacy of multiple anti-HIV drugs. Last, in vitro optimization studies on the dose- and time-dependent effects provide evidence that a safe treatment-regimen to exploit the anti-HIV efficacy of CyO2 may be possible in vivo. CyO2, a member of the cyclotide family, rapidly disrupts lipid membranes, [38] and its potency is evident on the negatively charged bacterial cell membranes [39]. Indeed, it is widely accepted that HIV-1 buds out from negative-charged microdomains of plasma membranes known as lipid rafts/caveolae, and these lipid rafts are highly enriched on the envelopes of the budding HIV-1 particles. Therefore, studies exploring the effects of cyclotides targeting these lipid rafts could be an evolving strategy to control the progression of AIDS.

In recent years, the discovery of natural products as anti-HIV agents [4,5], their utility in drug sensitization [52] and their ability to suppress the side-effects of cART [22,53,54] have shown encouraging results in vitro; however, their lack of in vivo efficacy has been a significant challenge due to their low bioavailability and systemic side effects at high concentrations. The cyclic cystine knot topology of cyclotides, which provides them exceptional stability against proteases and denaturation at low pH, address an essential attribute for a polypeptide drug candidate [55,56]. In this respect, the potential advantages of using this large family of phytopeptides as anti-HIV agents, either alone or as an adjunct to cART, should be further explored. 

To date, the database of cyclic proteins (http://www.cybase.org.au/) lists over 250 naturally occurring cyclotides that have been isolated from 57 plant species [56,57]. Over 20 cyclotides from at least eight different violaceous and rubiaceous taxa have been tested and exhibit some anti-HIV activity [27,35,58]. However, the molecular mechanism/s linked to their potent anti-HIV effects is not thoroughly elucidated. Our current findings, using both lymphocytic and monocytic cell lines (HIV IIIB H9, U1, HuT78, PM1 and U937) and using several different HIV-1 strains (HTLV-IIIB and LAV), clearly showed that CyO2 can disrupt the integrity of lipid membranes on both HIV-infected cells and infectious viral particles, suggesting the potential efficacy of cyclotides. However, despite their sequence similarities, it is possible that different cyclotide members may have utility in targeting different cellular membranes of HIV-1-infected cells or envelopes of different viral strains. In addition, depending on their sequence variations, different cyclotides may be able to target a variety of clinical strains/clades of the virus, as well. Thus, additional studies to fully evaluate the pore-forming mechanism of cyclotides as potent antiviral targets are clearly warranted. 

In this study, the prototypic bracelet cyclotide, CyO2 was chosen based on previous findings that CyO2 possesses powerful antibacterial activity [39], the availability of procedures for identification and extraction of CyO2 in our laboratories [45], and our past findings on its promising anti-HIV properties [44] and drug-sensitizing effects [45]. However, since the potent hemolytic and cytotoxic effects of micromolar (μM) doses of cyclotides (especially CyO2) have hampered their utility, our studies first optimized the therapeutic concentrations using hemolytic assays, which indicated that nanomolar (nM) doses of CyO2 can be used to inhibit HIV-1 infectivity. Indeed, hemolytic assays carried out in presence of human serum indicated that very few human RBCs were lysed with CyO2 concentrations of < 2.5 μM (Figure 2D).

With respect to the mechanism of antiviral effects of different cyclotides, the selective affinity for membranes rich in PE head-groups is associated with the bioactivity of different cyclotides [30,31,50]. The known anti-HIV activity of the cyclotide kB1 was shown to be due to the formation of pores that target membranes rich in phospholipids [30]. Interestingly, cyclotides in both Bracelet and Möbius subfamilies, as well as chimeric cyclotides (i.e., cyclotides that exhibit properties of both subfamilies) display anti-HIV activities [50]. Indeed, acyclic mutations introduced into circulins, cycloviolins, and kalata B1 (kB1) were shown to fully lose their antiviral activities [30,31]. Structural nuclear magnetic resonance (NMR) studies also clearly indicated a correlation between loop hydrophobicity of different cyclotides and their cytotoxicity. Recent studies have suggested that, compared to tricyclon A and several kalata cyclotides (kalata B1–B9), the positively-charged CyO2 has greater membrane affinity, leakage efficiency, and induction of membrane surface destabilization [55]. Additional attributes postulated to enhance CyO2 interactions with PE head-groups include structural characteristics such as the Lysine/Arginine side chain in loop 6, the Glutamic acid residue in loop 1, the placement of Lysine residues that encourage contact via electrostatic attractions and H-bonds, and finally, the close proximity of the hydrophobic and bioactive patches [38,39,49]. Therefore, the potent membrane disrupting properties of CyO2 may include a preferential attraction to anionic membranes, followed by insertion into the membrane and multimerization to form aqueous pores. However, a more thorough understanding of the targeting ability of CyO2 will be needed to fully elucidate its potent anti-HIV effects.

The selective ability of CyO2 to target specific membranes may enable it to achieve anti-HIV effects at non-toxic serum concentrations. Indeed, a threshold concentration of CyO2 is necessary for its multimerization and formation of stable conductive channels [38,39]. The current work showed that even short-term exposure to non-hemolytic doses CyO2 enables transient pore-formation on membranes that are rich in lipid-rafts, e.g., productively-infected cells and HIV-1 envelope. This may be a mechanistic explanation for the ability of CyO2 to selectively target the PE-enriched membranes of HIV-infected cells, as compared to uninfected cells. However, future studies are warranted to address the full mechanism(s) of action of CyO2 on both HIV-1-infected cells and infectious viral particles.

The current in vitro findings implicate a therapeutic window for the safe pre-clinical testing of CyO2 in animal models of HIV-1 infection [55]. Although the in vivo effects of cyclotides, especially CyO2, has not been thoroughly studied before, our collaborators have shown that less than 2 mg/kg dose of CyO2, given intravenously (i.v.) can be used safely in a mouse model, without significant toxicity or hemolytic activity [59,60]. This dose, when calculated according to the plasma concentrations achieved in mice, was found to be ~1.5 μM. Most interestingly, recent studies using another cyclotide, kB1 showed its efficacy in a mouse model of multiple sclerosis [61]. As much as a 20 mg/kg oral administration of kB1 was found to be non-toxic in these mice. Therefore, at physiologically safe concentrations, CyO2 may also provide a promising new target in anti-HIV drug research. 

The SYTOX-green assay results (Figure 3A–C) and the radiolabeled drug-uptake studies (Figure 3D) clearly showed that CyO2 can rapidly disrupt the membranes in HIV-infected cells, and increase the intracellular levels of ^3^H-SQV. The importance of this rapid effect of CyO2 is further underscored by findings that CyO2 pre-exposure for as little as 10 min enabled a 3–4 fold increase in intracellular ^3^H-SQV levels, and almost 5-fold higher drug levels were possible after 30 min exposure to CyO2. Similarly, the HIV-1 p24 ELISA studies documented that CyO2 can enhance the antiviral efficacy of multiple PIs (Figure 4). Indeed, numerous past studies have shown that the large size of peptidomimetics like SQV and NFV may severely compromise their entry into cells [18], which facilitates the selection and resurgence of drug-resistant HIV-1 clones [6,17]. The current findings implicate that CyO2 may augment the anti-HIV efficacy of multiple PIs, which could be a new direction in adjuvant approaches to the cART regimen in HIV-positive patients. 

Our novel findings suggest that CyO2 has a direct anti-HIV effect. Since the lipid envelope of HIV-1 particles are enriched with raft-like membrane constituents [15,43,51] we hypothesized that CyO2 may disrupt the integrity of viral particles. Congruent with this hypothesis, data obtained with VLPs showed that pre-exposure to low-dose CyO2 decreased the number of GFP-labeled cells (Figure 6A,B). Furthermore, the p24 ELISA results obtained with PM1 cells also showed that the CyO2-treated HTLV-IIIB virus had decreased ability to infect these lymphocytes (Figure 6C,D). Interestingly, in contrast to the single-round infection model (VLP), the effect of CyO2 on the infectious HTLV-IIIB model showed a more dramatic suppression, further implicating the potent ability of CyO2 in inactivating the infectious virus. Further corroboration of virus inactivation by CyO2 was evident from both ultracentrifugation studies that measured particle disruption (Figure 6A) and especially, from results obtained with the reporter line, TZM-bl [48]. These TZM-bl cells were engineered to overexpress HIV-1 receptors (CD4, CCR5 and CXCR4) and contain the firefly Luciferase gene under the control of HIV-1 LTR and have been frequently used as a highly quantitative HIV-infectivity assay [48]. Indeed, RLU data showed that the CyO2-pretreated virus completely loses its ability to infect cells (Figure 6C). Moreover, as compared to the effect of CyO2 on viral particle disruption (Figure 6B), the TZM-bl assay also indicated its potent suppressive effect on viral infectivity. Currently, there are no drugs available that target the infectious cell-free virions that are continuously being released from productively-infected cells, and the ability of CyO2 to inactivate the virus will be a promising approach in patients with uncontrolled viral load. 

Although previous studies have primarily focused on the membrane-active properties of CyO2, other investigators have suggested that cyclotides may also affect intracellular signaling mechanisms [61,62,63]. Indeed, it is well known that cellular transcription factors and viral transactivator (Tat) protein can activate HIV-1 LTR directed transcription [13,64]. Hence, to ascertain whether the anti-HIV effects of CyO2 may be due to its suppressive effects on provirus reactivation, two reporter cell lines that express GFP under the control of HIV-1 LTR, i.e., U937-VRX (Figure 5A) and J-Lat (9.2) (Figure 5B) were used. Analysis of GFP-positive cells demonstrated that CyO2 does not alter basal LTR function or affect the PMA-stimulated LTR function. These findings further corroborated that the lipid envelope of HIV-1 particles is most likely a target for CyO2, which prompted further investigations on its use in combination with anti-HIV agents that suppress viral entry.

The HIV-1 fusion inhibitor enfuvirtide (T-20) suppresses viral entry, and although very potent, it is usually kept as a ‘salvage therapy’ when patients present with drug-resistant HIV-1 strains and increasing viral load [10]. The work described here investigated whether the anti-HIV effects of CyO2 can enhance the efficacy of T20. Indeed, sub-toxic doses of CyO2 (0.5 μM) alone and in combination with T20 (4–100 nm) documented their potent effect when combined treatment was carried out in the PM1 cells (Figure 7D,E). Although T-20 alone was able to suppress p24 production by 3–5 fold, co-exposure to CyO2 totally abrogated p24 production; as evident within 3-days (Figure 7D) and 6-days (Figure 7E) post-infection. Thus, the two-pronged effect of CyO2, on both HIV-infected cells and infectious viral particles, can lead to significant enhancements in the effectiveness of multiple ARVs. 

Our initial studies provide a proof-of-concept to guide future exploration into the potential additive or synergistic effects of CyO2 with HIV-1 protease inhibitors and fusion inhibitors. Although SQV, NFV, and T-20 may not be the strongest contenders or most routinely used drugs in contemporary clinical settings, this research evaluated these drugs as an initial avenue to investigate the potential of these classes of ARVs. To this end, further studies using several newly approved PI drugs such as Darunavir and Amprevair may also provide valuable insight regarding the therapeutic benefits of adjunct CyO2 treatment. Furthermore, in addition to enfuvirtide (T-20), maraviroc is another entry inhibitor, where future studies conducted with CyO2 co-exposure may provide additional supporting evidence on the possible impact of CyO2 towards suppression of viral entry. Additionally, future studies could evaluate co-exposure of CyO2 with other classes of cARTs such as nucleoside reverse transcriptase inhibitors (NRTIs), non-nucleotide reverse transcriptase inhibitors (NNRTIs), integrase inhibitors (INSTIs) and chemokine receptor antagonists. Although these experiments were beyond the scope of the current research, future studies using multiple anti-HIV agents may indeed produce a valuable comparison of the effectiveness of CyO2 combination therapy across the different classes of ARVs and help discover the best candidates for additional testing. 

Ultimately, however, although our findings suggest that CyO2 is effective in increasing ARV entry into productively-infected cells and in the disruption of infectious viral particles, a formidable challenge with the treatment and cure of HIV-infected patients is the elimination of latent reservoirs, which persist despite cART [65]. Therefore, in light of the findings that, in addition to their membrane-active properties, cyclotides may have intracellular targets as well [61,62,63] it would be interesting to test the efficacy of CyO2 in combination with currently approved latency-reversing agents (LRAs). Indeed, investigations in this direction may expand the knowledge of how cyclotides could be used to facilitate the eradication of latent reservoirs and the suppression of disease progression in patients. 

## 5. Conclusions

**Figure 8:**
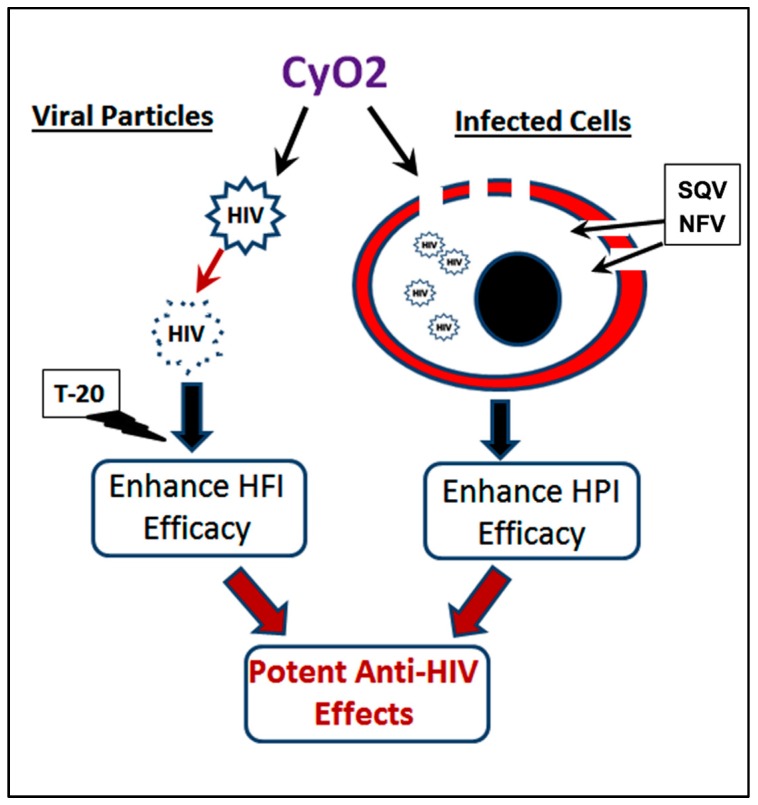
CyO2 targets both viral particles and HIV-1-infected cells. Membrane-targeting ability of CyO2 causes pore-formation in infected cells, which enhances the uptake of HIV-1 protease inhibitors, e.g., SQV and NFV. Furthermore, the ability of CyO2 to target the envelopes of viral particles suppresses their infectivity, which enables CyO2 to enhance the efficacy of an HIV-1 fusion inhibitor drug, T-20. Thus, this two-pronged effect of non-hemolytic doses of CyO2 may be a novel approach to augment the efficacy of multiple anti-HIV agents.

Thee pore-forming ability of subtoxic doses of CyO2 in productively infected cells and its ability to preferentially disrupt the lipid envelope of viral particles may be exploited to augment the efficacy of multiple ARVs (Figure 8). Our findings suggest that, at safe doses, this cyclotide may have potential as an adjuvant with multiple currently approved anti-HIV therapy, such as PIs and FIs. Importantly, since the lipid envelope of viral particles is a novel drug target, CyO2 may be used to suppress the infectivity of viruses that have become resistant to currently approved ARVs, as well. In this respect, future directives for studies with CyO2, and other cyclotides, should include in vitro serial passages of HIV-1 with increasing concentrations of different cyclotides; which will help delineate their mechanism(s) of bioactivity and their therapeutic potential. In addition, studies evaluating the clinical applications of CyO2, and other cyclotides, in pre-exposure prophylaxis or as topical microbicides, will provide new directions in antiviral research. Finally, investigating the potential of CyO2 as a natural compound to neutralize the circulating virus in vivo, may be of significant translational value in the clinical setting.
